# Supplementary material for: A Novel Catalytically Inactive Construct of Botulinum Neurotoxin A (BoNT/A) Directly Inhibits Visceral Sensory Signalling
Source: Toxins (Basel). 2024 Jan 7;16(1):30. doi: 10.3390/toxins16010030 (PMC10820156; doi:10.3390/toxins16010030)
Supplement: Supplementary file 1 [file toxins-16-00030-s001.zip › toxins-2777748-SI.pdf]

A novel catalytically inactive construct of botulinum neurotoxin A (BoNT/A) directly inhibits visceral sensory signaling

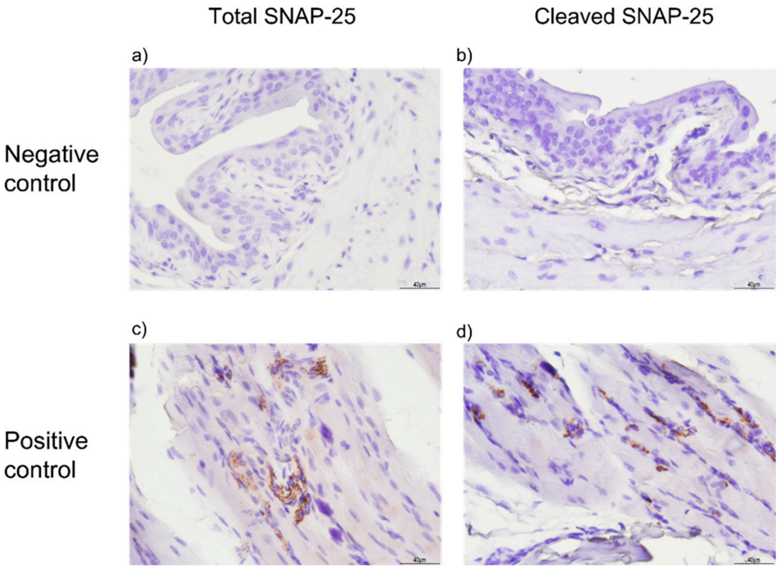

Figure S1. Negative and positive controls for total SNAP25 and cleaved SNAP-25 antibodies using skeletal muscle tissues sections from rats treated with aboBoNT-A.
